# Supplementary material for: Phylogeny of the Chinese Subgenera of the Genus Homoneura (Diptera, Lauxaniidae, Homoneurinae) Based on Morphological Characters
Source: Insects. 2022 Jul 22;13(8):665. doi: 10.3390/insects13080665 (PMC9331381; doi:10.3390/insects13080665)
Supplement: Supplementary file 1 [file insects-13-00665-s001.zip › Supplementary Files.pdf]

Table S1. The species studied

subfamily	Genus	Subgenus	Species
Lauxaniinae	*Minettia*	*Frendelia*	*Minettia* (*Frendelia*) *longipennis* (Fabricius, 1794)
Lauxaniinae	*Pachycerina*		*Pachycerina decemlineata* de Meijere, 1914
Homoneurinae	*Cestrotus*		*Cestrotus liui* Shi, Yang *et* Gaimari, 2009
Homoneurinae	*Dioides*		*Dioides incurvatus* Shi, Li *et* Yang, 2009
Homoneurinae	*Phobeticomyia*		*Phobeticomyia motuoensis* Li, Qi *et* Yang, 2020
Homoneurinae	*Prosopophorella*		*Prosopophorella yoshiyasui* Sasakawa, 2001
Homoneurinae	*Noonamyia*		*Noonamyia umbrellata* Shi *et* Yang, 2009
Homoneurinae	*Homoneura*	*Chaetohomoneura*	*Homoneura* (*Chaetohomoneura*) *disciformis* Shi, Wang *et* Yang, 2011
		Euhomoneura	*Homoneura* (*Euhomoneura*) *shatalkini* Papp, 1984
			Homoneura (*Euhomoneura*) *yanqingensis* Shi, Gao *et* Li, 2017
		Homoneura	*Homoneura* (*Homoneura*) *beckeri* (Kertész, 1900)
			Homoneura (*Homoneura*) *flavida* Shi *et* Yang, 2009
			Homoneura (*Homoneura*) *dorsacerba* Gao, Shi *et* Han, 2016
			Homoneura (*Homoneura*) *posterotricuspis* Gao, Shi *et* Han, 2016
			Homoneura (*Homoneura*) *acrostichalis* (de Meijere, 1915)
			Homoneura (*Homoneura*) *crassicauda* Malloch, 1927
			Homoneura (*Homoneura*) *picta* (de Meijere, 1904)
			Homoneura (*Homoneura*) *procerula* Gao *et* Yang, 2005
			Homoneura (*Homoneura*) *noticomata* Shi *et* Yang, 2014
			Homoneura (*Homoneura*) *trispina* Malloch, 1927
		Minettioides	*Homoneura* (*Minettioides*) *orientis* (Hendel, 1908)
		Neohomoneura	*Homoneura* (*Neohomoneura*) *nigrimarginata* Shi, Wang *et* Yang, 2011
			Homoneura (*Neohomoneura*) *tricuspidata* Shi *et* Yang, 2008
			Homoneura (*Neohomoneura*) *zengae* Shi *et* Yang, 2008

Table S2. Morphological dataset used for the analysis of the phylogeny.

Taxon	Characters
Minettia (*Frendelia*) *longipennis*	000000000000000000000000000000000000000000000000000000000000000<br>000000000000000000000000000000000000000000
Pachycerina decemlineata	010110100101110000100000112010100100110001001000000000000000000<br>11000000011100000101-----1---101000000100
Cestrotus liui	110001002001110111110101101010001100110121000000112300211011001<br>100000002010011010101110011---000011101100
Dioides incurvatus	110000002001110110101001111010000100110121000110112310211011001<br>110001001000110000100110011---010110100111
Phobeticomyia motuoensis	010000101001110010110001110010000100100021001010112310211011001<br>110000001010011111001000010010110110100100
Prosopophorella yoshiyasui	110002002001001110111011011000000100111121001001112310211011001<br>100000002110110110100000010110000011100100
Noonamyia umbrellata	010000101001000000101100112000001101110111000010112300211110111<br>100000001000010000100001010011000011110100
Homoneura (*Chaetohomoneura*) *disciformis*	010000000101100000000000011110110000001000201120100000000000000100<br>10000000200001110011020101010011011110?????
Homoneura (*Euhomoneura*) *shatalkini*	0110001010010000000000011111010012100100121000010001211100001000<br>110000000001010100110110010100000100100110
Homoneura (*Euhomoneura*) *yanqingensis*	0110000010010000000000011112010012110100021000010001111100001001<br>110000002000010100001000010100000110100110
Homoneura (*Homoneura*) *beckeri*	0100000102011001000101001111010000001001210010100000001000000000<br>111111101000110100110301110100010111100110
Homoneura (*Homoneura*) *flavida*	01000001020100010000000011111010000001001210010100000001000000000

	1111101020001101001013010101000101111100100
Homoneura (*Homoneura*) *dorsacerba*	01100000100100010000001101101000010010012100001011231110000 1000<br>1111111101101011101120101010000 0110100100
Homoneura (*Homoneura*) *posterotricuspis*	011000001001000100000011011010000100100121000010112311100001000<br>1111111201101111101120101010001 0110100100
Homoneura (*Homoneura*) *acrostichalis*	011100001001000100000011111011000010100121000010000000000000101<br>110002000110010100000011010000100010 1?????
Homoneura (*Homoneura*) *crassicauda*	011100001001000100000011111011000100100121000010000000000000100<br>110002000100010001100211110000010100 1?????
Homoneura (Homoneura) picta	010000001101100100000101011010100100100111001010112311211011100<br>1111111110000101000102011101000000 11100100
Homoneura (Homoneura) procerula	010000001101000100000101011010100100100111001010112311211011100<br>1111111001011010100120111010001000 0100100
Homoneura (Homoneura) noticomata	000100001000000100010000110001000000100111000010000000000000100<br>0000000010101111110011100101000000000100100
Homoneura (Homoneura) trispina	000000001010000100010001110001000000100121001010000000000000100<br>0000000020000110001001101101000101011 00100
Homoneura (Minettioides) orientis	011100001001000000000100111011000000000111000010000000000000110<br>1100020000011101010012010000000011111102100
Homoneura (Neohomoneura) nigrimarginata	011000001001000000000101111011000000100021011010001101200000100<br>1100000020111101001002010101011101111?????
Homoneura (*Neohomoneura*) *tricuspidata*	011000001001000000000000111011000000100021011010001101200000100<br>1100101020110101001001010101001001111 00100
Homoneura (*Neohomoneura*) *zengae*	011000001001000000000000111101100000010002101101000110120000010 0<br>1100000020110100101002010101001001111 00100
